# Calcarea-Ostrearum, a Study

**Published:** 1892-03

**Authors:** A. McNeil

**Affiliations:** San Francisco, Cal.


### CALCAREA-OSTREARUM, A STUDY.

A. MCNEIL, M. D., SAN FRANCISCO, CAL.

This drug was formerly called Calcarea-carbonica. But this name is a misnomer. It is not carbonate of lime properly speaking, but an impure carbonate prepared by triturating oyster shells, thus forming an animal preparation, not a chemical one.

Calcarea-ostrearum is an antipsoric of the purest type. Arsenicum and some others are applicable to acute diseases as well as to syphilis and sycosis, but this remedy is useful in the clearest cases of psora, or what is usually designated scrofula.

It is also according to Bœnninghausen an anti-sycotic.

We will better understand this invaluable remedy by considering its action on the blood. Every drug has one symptom which I think is best designated by the title "pivotal," as around this one all others seem to revolve. It might be described also as the always present symptom.

The pivotal symptom of Calcarea-ostrearum is the *leuco-phlegmatic constitution.* We recognize this by the patient's personal appearance. Only blonds are liable. In the adult much more frequently in the female we see a pale face, which is round and flabby, the form is full often to obesity, but the flesh is very soft.

He chills by the slightest cold air, which seems to go right through him. This constitution arises from the state of the blood. The red corpuscles are lessened in number while the white ones are increased, and these we know are prone to degenerate into pus corpuscles. The serum is also too abundant.

Now let us investigate as to the effects of this abnormal condition, which is such that it cannot supply the necessary constituents to build up the body, particularly in the young, so that school-children's headaches are often cured by it. Calc-phos., Natrum-mur., and Phosphoric acid are the remedies for the headaches of school-girls.

Leuco-phlegmatic people are subject to profuse discharges of mucus from all the mucous surfaces. So we see that the Calcarea child usually has fluent nasal discharge, so that the rude may speak of it as a "dirty-nosed young one." Women, and even young girls, have leucorrhœa, which, like all the catarrhal discharges of this drug, are bland and profuse. Puls. has a similar condition of the blood, and therefore similar profuse, bland mucous discharges.

The *vis medicatrix naturæ,* as we have seen, makes an effort to dispose of the surplus serum and white corpuscles of the blood by the catarrhal discharges, so she likewise does by an increase of the physiological discharge of blood. The menses are too early, too profuse, and last too long, and return on slight provocation, as from slight excitement. She also throws off the excess of serum by profuse sweats. The Calcarea patient sweats easily and profusely. We see on the infant when it falls asleep large drops which stand on the forehead and wet the pillow. The feet sweat, so as to feel as if stockings were damp. In hectic conditions we also have night-sweats. We differentiate from the various morbid perspirations which indicate Silicea by the latter being fetid or putrid, while those of Calcarea are either free from odor or smell sour. The Calc. patient always sweats easily and profusely, as we have seen, when asleep or on slight exertion.

We find the same tendency to get rid of the excessive serum and white corpuscles by suppuration. There is no remedy that controls suppuration more frequently. This occurs in the following manner: The leucocytes are going the rounds of the circulation, and the *vis medicatrix naturæ*, recognizing the evil which will result, impounds them in the glands, and thereby renders them innocuous in a measure. But collected together in this way, they may be likened to a foreign body which excites suppuration when imbedded in the tissues. Our remedy, when indicated, administered in a proper dose controls this morbid excess, and thus restores the enlarged and indurated glands to their normal condition, and checks the suppuration by removing the material which degenerates into pus. All the tissues are imperfectly nourished by the impoverished blood, thus the Calcarea patient is weak, mentally as well as physically, for the brain cannot do its work well when nourished only by this watery blood. We therefore see that thinking is difficult just as physical exercise is only performed with an effort. The patient feels as if he would lose his reason, or that others will think so. Like all weak creatures, he recognizes his weakness, and is anxious and restless, particularly as soon as the evening comes, so that he shudders with the dread he feels. He is despairing, being hopeless of getting well, and fears that he will die. He is therefore subject to delusion and mania of a timid character—is afraid of rats and mice, of fire, and of being murdered.

Owing to the poverty of the blood supplying the brain, he is attacked by vertigo, and the exertion of climbing, going upstairs, or even looking up, brings on an attack, so imperfectly is the brain nourished. Iodum also has vertigo from ascending, and also from looking up. He has a headache that begins on the occiput and spreads to the top of the head. With Gelsemium, Sanguinaria, and Silicea it begins in the same place, but goes to the forehead. Of course, this watery blood is favorable to the production of hydrocephalus and hydrocephaloid in infants.

Let us see how this drug affects them.

The Calcarea baby is fair, with a large head ; the fontanelles are large, as the bone is so poorly nourished ; the head sweats whenever he falls asleep, so that it stands in beads and wets the pillow ; the teeth are slow in coming, as they are literally starved, and their advent is attended by many troubles. Nature tries to get rid of the superabundant serum by diarrhœa as well as by the sweat we have mentioned. These diarrhœas and sweats are sour, as well as the vomited matter thrown up, while in its great rival, Silicea, all of these discharges and secretions are fetid and putrid.

The enlargement and suppuration of glands I have mentioned are more frequently met during the age of dentition, as that process makes heavy demands for material to build up the bone, teeth, and other tissues which the blood is unable to supply except of an inferior quality and deficient in quantity. And the leucocytes are intercepted in the glands, with the result before described.

The eyes, both in their mucous membranes and deeper tissues, often suffer from mal-nutrition. We therefore see ulcers on the cornea, conjunctivitis, and fistula lachrymalis, the indications for which are usually found in the constitutional peculiarities I have delineated. The same may be said of the ears. But there is one disease which is cured only by this drug, viz., hardness of hearing caused by the abuse of Quinine. I once made a brilliant cure of this life-long affliction with Calc$^{200}$. Of course it follows that with the increased serum in the blood that it is poor in albumen. Nature endeavors to overcome this by implanting a craving for eggs in the Calcarea patient.

Calcarea corresponds to an acid condition of the patient, so that he has a sour taste in his mouth, vomits sour substances, has sour sweats, diarrhœa, and sputa.

The pit of the stomach is swollen so as to form a tumor. This condition we find with nearly all the abnormal conditions as well as those of the digestive organs.

In the abdomen we find the same enlargement of the glands I have already pointed out. Enlargement and induration of the mesenteric glands in children having a psoric taint is frequent and dangerous. Of course these glands are seriously disabled in the performing of their duty in absorbing the nutritious portions of the food in the intestines, therefore we find great emaciation, a wrinkled and old look of the face, while, of course, the osseous system betrays the poverty of its nutrition by the slowness and difficulty with which the teeth are cut and by the large size of the head and potency of the fontanelles. In this condition you will have to consider the claims of Silicea, Iodum, Sulphur, etc. The Silicea patient has fetor of the secretions and discharges. It is often dark, and when fair has not that milky whiteness of the skin of Calcarea. The Iodum patient has black hair and eyes and eats often and large quantities, while it loses flesh all the time. The Sulphur patient kicks off the bed-clothes, as there seems to be an abnormal development of its animal heat, particularly in the feet, which are burning both objectively and subjectively, and the discharges from the bowels and bladder excoriate and turn red the parts they touch.

Next to the diseases of nutrition of infancy in importance come the diseases of the sexual sphere in women. The personality of the patient will frequently give you the guiding symtoms so that you will many times be able to select this remedy before your patient has stated her case. On inquiring you will find that she can scarcely stop menstruating, menses too early, too profuse, last too long, and return at any little excitement. The vaginal and uterine mucous membrane are like all those tissues elsewhere, throwing off profuse bland discharges. The pregnant woman, who is a Calc. patient, miscarries easily, as all her tissues are lax and weak. When she comes to nurse the some faulty nutrition clings to her. She secretes too much milk, the poor quality of which is shown by the infant's refusing to nurse, as does the baby whose mother requires Silicea.

In tubercular conditions of the lungs Calc. stands in the front rank along with Kali-carb., Lycopodium, Silicea, and Sulphur. It acts on the upper and middle third of the right lung ; with

Arsenic we have acute, sharp, fixed, or darting pain in apex and upper third of right lung. The chest is painfully sensitive to touch, resembling Cinchona with which this sensitiveness is so great that the patient cannot bear to be percussed or even auscultated. As from the other mucous membranes, the sputum is profuse; it tastes sweet, sour or salty. The cough is induced by playing on the piano, so that patient feels every note she strikes in the larynx. It is worse in the morning on rising and early in the evening. Of course the personality of the patient corresponds to that which belongs to Calc., viz., the leuco-phlegmatic constitution. Do not, I implore you, grow indifferent and careless in selecting the remedy because you have diagnosed consumption, even if the bacillus be present. But be as careful as you are when you cut within a line of the carotid, for, as in that case, life and death depend on your skill and care.

When we come to the limbs much of the usefulness of Calc. remains to be told. The infant is slow in learning to walk, as its muscles are soft, although they may be as large as if they belonged to an infant Hercules, for, as I have already pointed out, they are distended with water instead of being built up with good muscular tissue. In ulcers of the legs, which are so obstinate to surgical treatment, and the bad effects of apparent success are so striking, this drug takes the first place. I once had a case of ulcer of the leg of over twenty years' standing, in a woman fair, fat, and forty. During all of those years she was afflicted with the ulcer, or was subject to the most violent colic. When the sores were healed by external treatment, which had been done several times, then and then only the colic returned. With this drug she was cured of both. Diseases of the joints frequently require this drug, as the poorly-nourished bones, as well as the soft tissues break down into pus. The constitutional symptoms, rather than the local ones, decide. The softness of the bones, owing to their being so poorly nourished by the watery blood, shows itself in bow legs. The feet and legs feel as if she had on cold, damp stockings. This is a symptom of vital importance, and will serve to lead you to the right selection many times.

As was to be expected from the poorly-nourished tissues, the nervous system shows the weakness under which it labors. The epilepsy indicating Calc-ost. is preceded by a feeling as of something running through the arms or down through the abdomen into the feet. They are worse at night, during the equinoxes, and the full moon. In insomnia, arising from the same thought perpetually recurring, give Calc-ost.

In eruptions, such as eczema, when the glandular system is involved, dentition slow, and the other manifestations I have pointed out, Calc. will cure.

It is complementary to Bell., and follows well after Lycop., Nitric-ac., and Sulphur, but should not precede the two latter.

